# Female mate choice of male signals is unlikely to promote ecological adaptation in *Enchenopa* treehoppers (Hemiptera: Membracidae)

**DOI:** 10.1002/ece3.3817

**Published:** 2018-01-22

**Authors:** Kasey D. Fowler‐Finn, Joseph T. Kilmer, Daniel C. Cruz, Rafael L. Rodríguez

**Affiliations:** ^1^ Behavioral & Molecular Ecology Group Department of Biological Sciences University of Wisconsin‐Milwaukee Milwaukee WI USA

**Keywords:** adaptation, ecological speciation, vibrational signal

## Abstract

A key question in speciation research is how ecological and sexual divergence arise and interact. We tested the hypothesis that mate choice causes local adaptation and ecological divergence using the rationale that the performance~signal trait relationship should parallel the attractiveness~signal trait relationship. We used female fecundity as a measure of ecological performance. We used a species in the *Enchenopa binotata* treehopper complex, wherein speciation involves adaptation to novel environments and divergence in sexual communication. We used a full‐sibling, split‐family rearing design to estimate genetic correlations (*r*_G_) between fecundity and signal traits, and compared those relationships against population‐level mate preferences for the signal traits. Animal model estimates for *r*_G_ between female fecundity and male signal traits overlapped zero—rejecting the hypothesis—but could reflect sample size limitations. The magnitude of *r*_G_ correlated with the strength of the mate preferences for the corresponding signal traits, especially for signal frequency, which has the strongest mate preference and the most divergence in the complex. However, signal frequencies favored by the population‐level mate preference are not associated with high fecundity. Therefore, mate preferences do not appear to have been selected to favor high‐performance genotypes. Our findings suggest that ecological and sexual divergence may arise separately, but reinforce each other, during speciation.

## 1 INTRODUCTION

Speciation is broadly thought to require divergence in ecological and sexual traits. Specialization on different resources brings performance trade‐offs that disfavor hybridization (Coyne & Orr, 2004; Nosil, 2012); and, divergence in traits such as ornaments, mate preferences, and genitalia yields direct reproductive isolation (Andersson, 1994; Coyne & Orr, 2004; West‐Eberhard, 1983). Together, divergence in these two dimensions results in distinct forms with independent gene pools.

It is straightforward to view ecological divergence as a product of divergent natural selection acting on populations adapting to different environments (Coyne & Orr, 2004; Nosil, 2012; Rundle & Nosil, 2005; Schluter, 2001, 2009). However, natural selection seems incapable of accounting for a key feature of speciation: Sexually selected traits often have much faster rates of diversification and elaboration than naturally selected traits, often being the only or the most useful diagnostics between closely related species (e.g., Andersson, 1994; Cocroft, Rodríguez, & Hunt, 2008; Coyne & Orr, 2004; Eberhard, 1985; Mendelson & Shaw, 2005; Safran et al., 2012; Seddon, Merrill, & Tobias, 2008; Seddon et al., 2013; Wells & Henry, 1998; West‐Eberhard, 1983, 2014).

It is also straightforward to view sexual divergence as a product of divergent sexual selection (Fisher, 1958; Higashi, Takimoto, & Yamamura, 1999; Kirkpatrick & Ravigné, 2002; Pomiankowski & Iwasa, 1998; West‐Eberhard, 1983). Sexual selection is stronger and more constant than natural selection (Hoekstra et al., 2001; Kingsolver et al., 2001; Svensson, Eroukhmanoff, & Friberg, 2006; West‐Eberhard, 1983). And, as noted above, sexual traits are often the most differentiated features among closely related species. Accordingly, sexual selection makes stronger contributions to sexual isolation than natural selection (e.g., Boul, Funk, Darst, Cannatella, & Ryan, 2007; Claridge, Den Hollander, & Morgan, 1985, 1988; Funk, Cannatella, & Ryan, 2009; Gray & Cade, 2000; Martin & Mendelson, 2016; Masta & Maddison, 2002; Sota & Tanabe, 2010). But, to fulfill the above speciation requirement, sexual selection would also need to create ecological differences among diverging populations to arrive at fully distinct forms.

Thus, a key question in speciation research is whether and how the interplay between natural and sexual selection contributes to ecological and sexual divergence (Kopp et al., 2018; Maan & Seehausen, 2011; Riesch et al., 2017; Safran, Scordato, Symes, Rodríguez, & Mendelson, 2013; Seehausen et al., 2014). The hypothesis that mate choice—a major cause of sexual selection—also produces local adaptation and ecological divergence describes one such potential interplay. According to this hypothesis, the very power of sexual selection discussed above is what contributes to the rapid creation of both sexual and ecological differences between diverging populations.

The rationale for the hypothesis that mate choice promotes local adaptation and ecological divergence is as follows: When sexual ornaments are costly, mate preferences for individuals with attractive displays would favor those individuals better able to acquire and allocate resources to the display. Selection favoring individuals with such displays would therefore also favor high‐condition, locally adapted individuals, thereby promoting local adaptation (Byers, Hebets, & Podos, 2010; van Doorn, Edelaar, & Weissing, 2009; Jennions, Møller, & Petrie, 2001; Lande & Kirkpatrick, 1988; Lorch, Proulx, Rowe, & Day, 2003; Pomiankowski & Møller, 1995; Proulx, 1999; Rowe & Houle, 1996; Tomkins, Penrose, Greeff, & LeBas, 2010; Wilkinson & Taper, 1999) . Mate choice on different environments could then promote differential local adaptation and hence ecological divergence (Cocroft et al., 2008; Lorch et al., 2003).

There is mixed support for this hypothesis. Sexual displays only sometimes show the predicted elevated levels of condition‐dependence (Cotton, Fowler, & Pomiankowski, 2004; cf. Koch, Josefson, & Hill, 2017). And, artificial selection experiments in which sexual selection is allowed only sometimes result in enhanced adaptation to novel environments (Coyne & Orr, 2004). A potential weakness of some of these tests is not taking into account that only a subset of sexual traits is expected to evolve high levels of condition‐dependence—for example, because of variation in their scaling with body size and in their degree of sexual dimorphism (Bondurianky & Rowe, 2005; Bonduriansky, 2007; Eberhard, Rodríguez, & Polihronakis, 2009). An additional problem may lie in the use of body condition as a measure of adaptation to an environment. Condition—the resources acquired by an individual that can be allocated to any one function or trait (Hunt, Bussière, Jennions, & Brooks, 2004)—should covary with performance on local environments. However, even when sexual displays are costly, they may mainly reflect the ability to operate at high levels of performance for relatively brief periods of time, which may be unconnected from the size of the resource pool available (Clark, 2012).

We consider that to relate mate choice of ornaments to local adaptation, the key is to focus on the fecundity of the choosing females and their daughters. This is a version of the hypothesis that male sexual ornaments are selected to indicate the quality of the daughters that males would produce if accepted as mating partners (Trivers, 2002; cf. Miller & Moore, 2007). The key question is therefore whether female fecundity covaries genetically with male ornaments. We do not hold that daughter fecundity will outweigh the attractiveness and/or viability of male offspring or other sources of selection on mate choice (Kokko, Brooks, McNamara, & Houston, 2002). On the contrary, we consider that sexual selection on mate choice may often be predominant (Prum, 2012, 2017; West‐Eberhard, 1983, 2014). However, for the hypothesis that mate choice promotes local adaptation and ecological divergence, it does seem to us to be the most relevant measure.

We therefore used female fecundity as a measure of the ecological performance of different genotypes to test the hypothesis that mate choice causes local adaptation. We generated predictions according to the rationale that there should be a relationship between genetic variation in performance and advertisement signals, and that the performance~signal trait function should parallel the attractiveness~signal trait function. We note that mate choice may promote divergent ecological adaptation without preferences for ornaments, if females focus directly on ecological traits (Byers & Waits, 2006; Reinhold, 2004). We focus on the widespread scenario of mate choice of advertisement signals (Andersson, 1994).

We list the predictions that arise from the above rationale in Table 1. Prediction (i) is a prerequisite for testing all the other predictions, rather than a logical part of the hypothesis. Predictions (ii)–(ix) articulate the rationale. Predictions (viii) and (ix) refer to comparative tests with closely related species—we did not test them because of lack of support for the preceding predictions, but we list them here for completeness and to encourage further studies.

**Table 1:** Predictions of the hypothesis that mate choice of male sexual ornaments promotes local adaptation and ecological divergence. Prediction (i) is a prerequisite for testing the hypothesis, more than a logical requirement. Predictions (ii)–(ix) articulate the rationale that there should be a relationship between genetic variation in performance and signal traits, and that this relationship should be parallel to the function relating attractiveness to those signal traits (see text). We state the predictions in general, and we also refine them with information about the mating system and mate preferences of our study species, a member of the *Enchenopa binotata* complex of treehoppers (Cocroft et al., 2008, 2010; Rodríguez et al., 2006). We also summarize the results of our experiment indicating the presence or absence of support for the predictions (see Section 3)

General predictions	Specific predictions for *Enchenopa*	Results from present study
(i) There should be genetic variation in female fecundity and in male signal traits	Same	Support
(ii) There should be a genetic correlation (*r*_G_) between female fecundity and a male signal trait	Same	Weak rejection
(iii) *r*_G_ should be strongest for the signal trait with the strongest mate preference	*r*_G_ should be strongest for signal frequency	Weak rejection
(iv) *r*_G_ should be strongest for the most distinctive signal trait among closely related species	*r*_G_ should be strongest for signal frequency	Weak rejection
(v) The function relating genetic variation in female fecundity to genetic variation in male signals should have the same shape as the function relating attractiveness to signal traits at the population level	The fecundity~signal frequency genetic function should be hump‐shaped, as the female mate preference function for signal frequency^a^	Reject
(vi) The highest point in the fecundity~signal trait function should correspond to the preferred value for the signal trait in the population (i.e., to the peak of the mate preference)	Genotypes with the highest fecundity should have signals with frequencies of ca. 318 Hz^b^	Reject
(vii) The peak of the mate preference should be narrow around signal trait values associated with high performance	Same	Reject
(viii) Among closely related species in different environments, different signal features (or different combinations of signal features) should correspond to high performance	Same	Not tested
(ix) Among closely related species in different environments, the signal features or signal feature combinations that correspond to high performance should be favored by the mate preferences of each of those species	Same	Not tested

a For the other signal traits examined here, the fecundity~signal length and the fecundity~signal number genetic functions should be hump‐shaped; and the fecundity~pulse number genetic function should be linear and rising with higher pulse numbers.

b For the other signal traits examined here, genotypes with the highest fecundity should have the following: whine lengths of ca. 0.5 s, bouts of ca. 7 signals, and ca. 7 pulses.

We tested the predictions with a species belonging to the *Enchenopa binotata* complex of treehoppers (Hemiptera: Membracidae). We used a quantitative genetics experiment to examine the genetic relationship between female fecundity and male signal traits. We then used playback experiments to describe population‐level female mate preferences for those signal traits.

The *E. binotata* complex is a clade of plant‐feeding insects that is widely distributed across eastern North America, with each treehopper species occurring on its own host plant species (Cocroft et al., 2008; Wood, 1993). *Enchenopa* communicate with plant‐borne vibrational signals, and signal variation across the complex is mostly associated with differences in host plant species, rather than geographic distance (Cocroft, Rodríguez, & Hunt, 2010). Thus, signal divergence in the complex has occurred through changes in selection on signals associated with the colonization of novel host plants. Sources of selection on signals that vary across host plants include divergent mate preferences and plant signal‐transmission properties (McNett & Cocroft, 2008; Rodríguez, Ramaswamy, & Cocroft, 2006). Of these, mate preferences seem to make the stronger contribution to realized mate choice decisions and, consequently, to selection on signals (Rodríguez et al., 2006; Sullivan‐Beckers & Cocroft, 2010). Pair formation in *Enchenopa* involves male–female duets (Cocroft & Rodríguez, 2005; Cocroft et al., 2008). Males initiate the duet by producing advertisement signals, and if a female finds a male's signals attractive, she signals back, prompting him to search for her on the plant (Rodríguez & Cocroft, 2006). Whether a female responds to a male's signals influences the likelihood of her mating with him. Females thus express their mate preferences in selective duetting with males (Rodríguez, Sullivan, & Cocroft, 2004; Rodríguez et al., 2006). The strongest mate preferences in the *E. binotata* complex are for male dominant signal frequency (Rodríguez et al., 2006), and this is the most distinctive signal trait among the members of the complex (Cocroft et al., 2010); consequently, the strongest genetic correlation (*r*_G_) should be between fecundity and signal frequency (Table 1). Similarly, detailed study of the shape of female mate preferences (Rodríguez et al., 2006; results of current study) allows us to specify the shape that the fecundity~signal trait functions should have for different signal traits, and where along variation in different signal traits those functions should peak (Table 1).

We also examined corollary factors that could influence the ability to test the predictions. For *r*_G_ to exist between female fecundity and a male signal trait, there should be some genetic variation in fecundity and the signal trait (prediction i). But, genetic variation may be lower in signal traits with strong mate preferences that exert stronger selection. We therefore tested for a relationship between the strength of mate preferences and the amount of genetic variation in the corresponding signal traits; and, we also tested for a relationship between the amount of genetic variation in signal traits and the magnitude of *r*_G_ between female fecundity and the signal traits.

## 2 MATERIALS AND METHODS

Most of the species in the *E. binotata* complex have not been formally described (Hamilton & Cocroft, 2009), but can be recognized by the host plants they use and the signals of the adult males. We worked with the species that lives on *Ptelea trifoliata* host plants (Rutaceae). We kept voucher specimens in 75% ethanol in the Rodríguez laboratory collection.

### 2.1 Experiment 1: Quantitative genetics of female fecundity and male signals

To test for a correlation between the signals and fecundity of siblings—between brothers’ signals and sisters’ fecundity—we used a full‐sibling, split‐family rearing design (Lynch & Walsh, 1998). We established full‐sib families from mated females collected in the field (at the University of Missouri Greenhouse grounds, Columbia, Missouri) in August 2012–2014. *Enchenopa* females mate only once (Sullivan‐Beckers & Cocroft, 2010; Wood, 1993), so offspring from a field‐collected mated female are full‐sibs.

We placed each female on her own potted *P. trifoliata* plant for egg‐laying during the late summer and fall (we acquired the plants from a local native plants nursery), at an outdoor facility at the UWM Biological Sciences Greenhouse. The eggs overwintered on the plants, and the nymphs hatched the following spring. When the nymphs reached the second instar, we split them into two replicate rearing plants per family, on which they remained until adulthood. When the nymphs molted to adults, we further split each replicate to keep the males and females on separate plants to prevent females from mating and becoming sexually unresponsive.

We recorded the advertisement signals of the males when they reached sexual maturity, ca. 2–3 weeks after the adult molt (vibrational recording procedure below). And, we used vibrational playbacks to describe female mate preference functions ca. 2 weeks later, when they reached maturity (playback procedure below).

We then paired the females with randomly chosen, unrelated, field‐collected males, placing each pair on a potted host plant. (As *Enchenopa* females mate only once, the fecundity of experimental females could not be assessed across more than one male; Sullivan‐Beckers & Cocroft, 2010; Wood, 1993.) The plants were covered with a screen cage to prevent the treehoppers from flying away while allowing the male and the female to interact freely. Note that this design confounds variation in the fecundity of each treehopper female with variation due to the male with which she was paired and with variation due to the host plant on which she was placed. However, the key parameter of fecundity for each full‐sib family was assessed with replication across host plant individuals. We allowed the females to lay eggs until they died in the fall with the first frost. Finally, we counted the eggs laid by each female as an estimate of her fecundity and ecological performance. We note that aspects of fitness other than fecundity (such as growth rates and survivorship to the adult stage) may be important. However, fecundity refers most directly to the fitness of the dams rather than to the fitness of their offspring, keeping the focus on the fitness consequences of mate choice for the individuals exerting that mate choice (Wolf & Wade, 2001).

#### 2.1.1 Male signals

Mate‐searching *Enchenopa* males move from one plant to another, signaling on each plant until they receive a response from a female (Cocroft et al., 2008). They also tend to signal when placed on a plant stem in the lab, and we took advantage of this behavior to record them. We placed males individually on a potted host plant in the lab. If a male did not start singing within 2 min, we played back a primer to help induce signaling. This primer consists of a male–female duet, and does not change the signaling behavior, other than encouraging a reluctant male to start signaling. We measured temperature near the recording plant to the nearest 0.5°C.

*Enchenopa* vibrational signals are transmitted as bending waves along plant substrates (Cocroft & Rodríguez, 2005). Recording these signals requires measuring the movement of the plant surface near the insects. We used a portable laser Doppler vibrometer (Polytec PLV‐100; Polytec Inc. Auburn, MA, USA). This high‐sensitivity method allows monitoring vibrational signals without contacting the plant, preventing any alteration of plant signal‐transmission properties, and is well suited for the low‐amplitude signals used by *Enchenopa*. We focused the beam of the laser on a piece of reflective tape (ca. 2 mm2) secured to the stem of the recording plant. We sent the laser signal through a high band pass filter set to 60 Hz (40–4,000 Hz, Krohn‐Hite 3202; Krohn‐Hite Corp., Brockton, MA, USA) to an iMac computer through a USB audio interface (Edirol UA‐25; Roland, Corp. Hamamatsu, Japan). We recorded the signals with the program AUDACITY (v. 1.2.5; http://audacity.sourceforge.net/) at a sampling rate of 44.1 Hz.

We isolated the recording setup (the potted plant and laser vibrometer) from noise due to building vibrations as follows: The plant and laser were placed on shock‐absorbing sorbothane pads (Edmund Scientifics, Tonawanda, NY, USA) on top of a heavy iron plank (135 kg) that rested on partially inflated bicycle inner tubes on top of a heavy table that stood on vibration damping pads (model 3291‐22‐PM‐50; Polymer Dynamics, Inc., Allentown, PA, USA).

**Figure 1:**
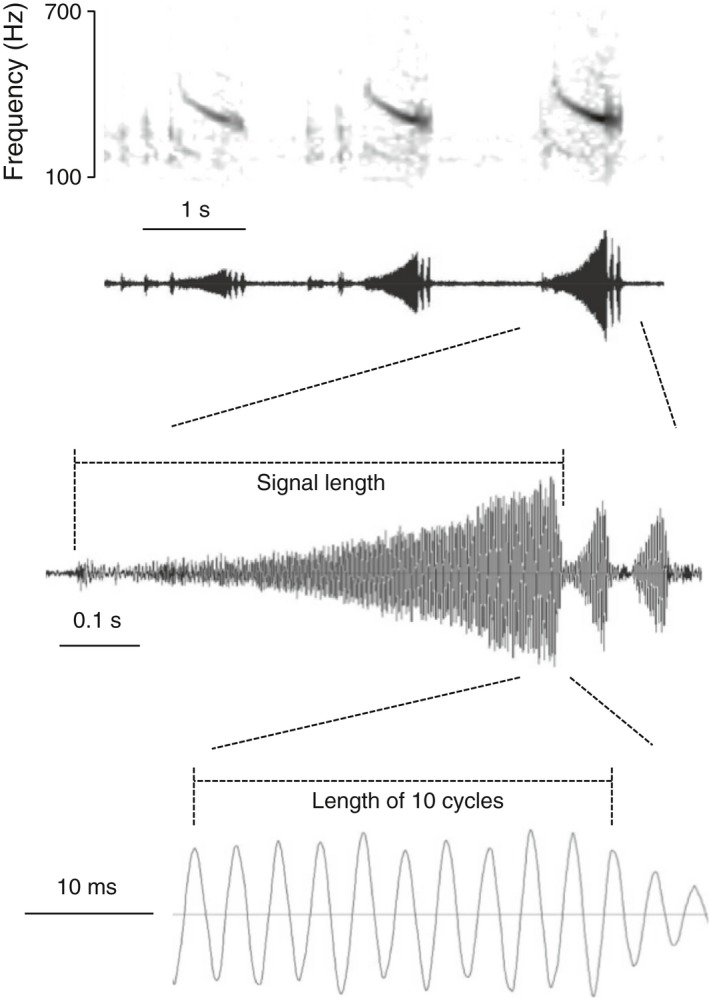
Spectrogram (top) and waveform of the signal bout produced by an *Enchenopa* male. Note that the signal consists of a pure tone that sweeps slightly downwards in frequency, followed by pulses. The spectrogram is for illustrative purposes; we took all measurements from the waveforms. We took the following measurements: the number of signals in the bout; the length of the signal; the number of pulses at the end of the signal; and the dominant frequency of the signal, calculated from the length of 10 cycles at the point of highest amplitude in the signal

For our analysis, we measured four signal traits that are associated with mate preferences of varying strength in our study species (Rodríguez et al., 2006). For the hypothesis, the most relevant signal trait is dominant frequency (Figure 1): It has the strongest mate preference and is the most distinctive signal trait among species in the complex (Cocroft et al., 2008, 2010; Rodríguez et al., 2006). The other signal traits that we measured were as follows, in decreasing order of the strength of the mate preferences: signal length, number of pulses, and number of signals (Figure 1).

*Enchenopa* males produce signals in bouts, along which signal features vary slightly (Cocroft et al., 2010). We standardized our measurements with a landmark position on a signal bout: the third signal of the second bout produced by a male, or the closest to this signal as possible (e.g., the second signal if a male produced only two signals in his second bout, or the third signal of the first bout if a male produced only one bout). We took all measurements from the signal waveforms in AUDACITY (Figure 1).

Signal dominant frequency was influenced by temperature; we therefore standardized all measurements to 23.5°C before the analyses described below (using the slope of the regression on temperature). The other signal traits were not influenced by temperature (*p *≥* *.25).

#### 2.1.2 Female fecundity

**Figure 2:**
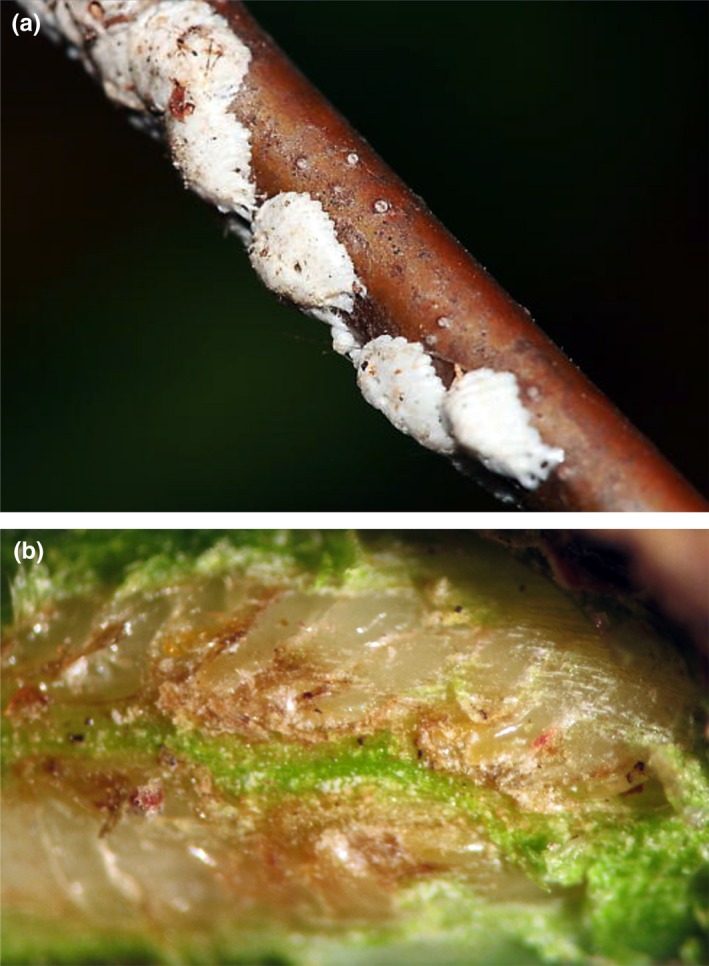
Eggs laid by an *Enchenopa* female. (a) Egg masses, each covered with a sculptured waxy coating. (b) Eggs revealed by removing the waxy coat and the thin layer of bark on the plant stem

*Enchenopa* females deposit their eggs in masses of ca. 10 eggs each, inserting each egg horizontally under the bark of the stem of the plant, and cover each mass with a coating of wax (Figure 2). This allowed us to easily identify all the egg masses laid by each female. We examined the masses under a dissecting microscope, using a scalpel to carefully scrape away the waxy coating and the first layer of bark to reveal the eggs and count them (Figure 2).

### 2.2 Experiment 2: Population‐level female mate preferences

We described univariate mate preferences for each of the above signal traits to compare each one with how the signal traits relate to variation in female fecundity. Differences in the shape and strength of the preferences give additional leverage to test hypotheses about their contribution to signal evolution and speciation (Rodríguez et al., 2006, 2013).

Mate preferences are representations of the attractiveness of signals according to their features (Kilmer et al., 2017). Analyzing mate preferences requires assessing attractiveness across a relevant range of signal trait values, that is, they are function‐valued traits (Kilmer et al., 2017). We used vibrational playback with synthetic stimuli resembling male advertisement signals. We placed each female on the stem of a potted playback plant and allowed her to acclimate for 2 min. We presented the stimuli through a piezoelectric stack connected to the stem of the plant, driven by a piezoelectric controller (Thorlabs, Newton, NJ, USA). We delivered stimuli at an amplitude of 0.15 mm/s. We controlled and created the stimuli with custom scripts in MATLAB (v. 7.5.0; The Mathworks, Inc., Natick, MA, USA) (scripts available upon request). We describe the preferences across stimulus values spanning 95% of the range of the population around the mean (i.e., mean ± 2 *SD*) for the signal trait, keeping the other features of the stimuli set to the mean of the population—this includes six signals/bout for all stimuli, except those for the signals/bout preference.

We obtained mate preferences with females from the 2012 rearing experiment. For each female, we first played back a recording of a live male to check her sexual receptivity. If she did not respond, we gave her another 2 min and tested her again, and if she continued to be unresponsive, we returned her to her rearing plant for testing on a subsequent day. If a female was responsive, we presented her with playbacks (in random sequence) to describe her preference for signal frequency and for signal length (in random order, with 10 min separating the playbacks for each preference). We recorded the playbacks and the females’ responses with the laser vibrometer and AUDACITY as per above. We were only able to conduct playbacks as above to describe the mate preferences for signal frequency and signal length. We did not have time to run playbacks for the other signal traits. We therefore used the data from a prior study with females from the same population (Rodríguez et al., 2006) for the preferences for signals/bout and pulse number.

Our assay of preference is based on the natural duetting behavior of *Enchenopa* females (see above). Females duet with artificial stimuli just as they do with live males, allowing for fine‐scale analysis of their mate preferences (e.g., Fowler‐Finn & Rodríguez, 2012a, 2012b, 2013; Rodríguez et al., 2006, 2013). We noted the number of responses that females produced in response to the stimuli. We then averaged across replicates (split‐families) and families for each stimulus to generate the population‐level response data. For the preference for signals/bout, rather than the number of responses, we used the percentage of the females tested that responded to the stimuli (because an increase in the number of responses with the number of signals/stimulus bout might simply reflect the opportunity to respond, rather than a preference for more signals in a bout).

We used the program PFunc (Kilmer et al., 2017) to generate preference functions. This program fits nonparametric cubic splines to the female response~stimulus feature data, and avoids any assumptions about the shape of the functions other than some level of smoothness (Kilmer et al., 2017; Schluter, 1988). To compare the preferences for different signal traits, we scaled the preferences produced by PFunc to the same maximum value (=1), by dividing by the maximum value for each preference. We then compared the mate preferences against the plots of the relationship between genetic variation in female fecundity and the corresponding signal traits (see below).

We estimated the strength of the mate preferences (the degree to which attractiveness changes over signal trait values) as the ratio of the standard deviation of female response across the preference function and the mean female response (Kilmer et al., 2017).

### 2.3 Data analysis

#### 2.3.1 Testing predictions (i) and (ii): Heritabilities and genetic correlations

To estimate heritabilities, we only used families with data for at least two individuals/replicate (Table 2) for each trait concerned. This criterion yielded *n *=* *16 families for the estimate of heritability in female fecundity; and *n *=* *30 for the estimate of heritability in male signals. Similarly, to estimate *r*_G*,*_ we only used families with data for at least two individuals/replicate/sex (Table 2) for the two traits. This criterion yielded *n *=* *16 families. This small sample size was a function of the difficulty of obtaining the full data for enough individuals in each replicate and family, which required keeping alive not only the males through signal recording but also the females through mating and the end of egg‐laying well into the Fall.

**Table 2:** Sample sizes for the families and split‐families (replicates) of *Enchenopa* treehoppers included in our estimates of trait heritabilities and *r*_G_

	Signals	Fecundity	Correlations	
			Signals	Fecundity
*n* within families				
Mean	12	6	12	6
Range	4–23	5–9	4–18	5–9
*n* within replicates				
Mean	6	3	6	3
Range	2–13	2–6	2–12	2–6

We used the animal model, implemented in R using the MCMCglmm package (Hadfield, 2010; Wilson et al., 2010). To represent the full‐sib, split‐family design in terms of the animal model, we coded pedigrees with one sire and one dam per family, with no relatedness among sires and dams. To examine whether the priors influenced the outcome of the model, we ran the analysis with three different ratios: with even priors (divided equally among individual, replicate, and residual effects); with priors biased toward the animal term (90% animal, 5% replicate, 5% residual); and with priors biased heavily toward the residual term (5% animal, 5% replicate, 90% residual). In all cases, we used low belief in the priors. We used chain lengths of 1,000,000 iterations, sampling every 1,000, with a burn‐in of 500,000. All autocorrelation values were <.005 by the end of the runs for the heritability estimates, and ≤0.01 for the genetic correlation estimates. We report estimates with 95% confidence intervals (CIs) and their posterior distributions. Note that our full‐sib, split‐family rearing design yields estimates of broad‐sense heritability as they may include nonadditive components such as dominance variance and maternal effects. We also report the amount of genetic variation as a coefficient of variation, following Houle (1992), but using the notation CV_genetic_ rather than Houle's CV_A_ to indicate that our estimates of genetic variance may include nonadditive components. We calculated CV_genetic_ thus: CV_genetic_ = 100 √(variance estimate)/mean.

We also estimated *r*_G_ with another method. We calculated the Pearson's correlation between family median values for female fecundity and male signal traits in JMP (v. 7.0.1; SAS Institute, Cary, NC, USA). To obtain these values, we first calculated the median for each split family and then obtained the median of those values for each family.

#### 2.3.2 Testing predictions (iii)–(vii): Comparing the fecundity~signal trait relationship with mate preferences

We used Pearson's correlations to assess the relationship between the strength of mate preferences and the absolute value of *r*_G_ between female fecundity and the signal traits. With *n *=* *4 signal traits, this correlation would have to be of very large magnitude to be statistically significant. We therefore focused on its effect size.

We overlaid scatterplots showing family median values and the mate preference function splines. The linear or curvilinear shape of these preferences was established in prior work (Rodríguez et al., 2006) and confirmed here. We tested for curvilinearity in the fecundity~signal trait relationships by fitting quadratic regressions.

### 2.4 Corollary analyses

We used Pearson's correlations to assess the relationships between the strength of mate preferences and the amount of genetic variation in the corresponding signal traits. We also used Pearson's correlations to determine the relationship between the amount of genetic variation in a signal trait and the magnitude of *r*_G_ between female fecundity and the signal trait. As above, we focused on the effect size of these correlations.

## 3 RESULTS

**Figure 3:**
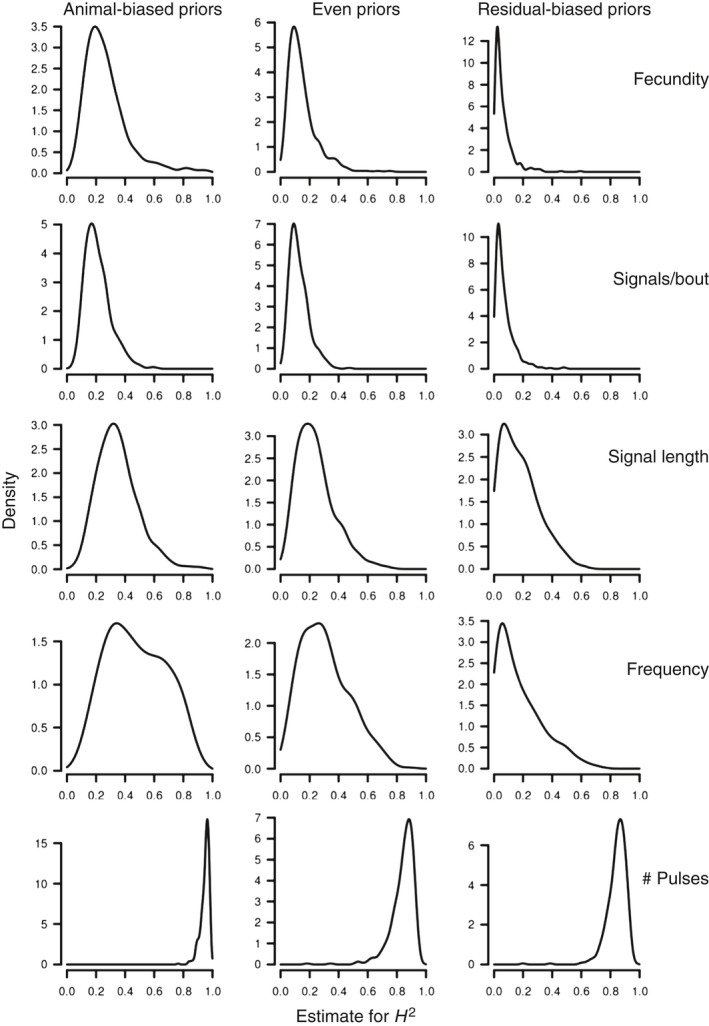
Posterior distributions of the heritability estimates for female fecundity and male signal traits in *Enchenopa* in our rearing experiment, with the different priors used in the animal model

We found heritability of small effect size in fecundity, and of small‐to‐large effect size across signal traits (Table 3). The different priors did not substantially influence the heritability estimates for female fecundity and most male signal traits, except for dominant frequency and signal length (Table 3; Figure 3). The key signal trait (dominant frequency) had heritability of low‐to‐medium effect size. This met the requirement outlined in prediction (i), and we therefore proceeded to test the other predictions (Table 1).

**Table 3:** Animal model estimates of broad‐sense heritability (with 95% confidence interval) and CV_genetic_ in *Enchenopa* female fecundity and male signal traits (Figure 1)

	Animal‐based priors		Even priors		Residual‐based priors	
	*H* ^2^	CV_genetic_	*H* ^2^	CV_genetic_	*H* ^2^	CV_genetic_
Females						
Fecundity	0.21 (0.05–0.60)	48.0	0.07 (0.02–0.36)	31.3	0.02 (0.006–0.18)	15.8
Male signals						
Frequency	0.33 (0.14–0.83)	9.1	0.16 (0.05–0.64)	6.7	0.02 (0.006–0.50)	2.1
Length	0.32 (0.12–0.63)	2.5	0.18 (0.03–0.50)	2.1	0.02 (0.004–0.43)	0.5
# pulses	0.97 (0.89–0.99)	25.0	0.88 (0.69–0.94)	16.4	0.87 (0.73–0.94)	9.1
# signals/bout	0.15 (0.06–0.38)	34.1	0.09 (0.03–0.27)	32.6	0.03 (0.004–0.20)	30.8

**Figure 4:**
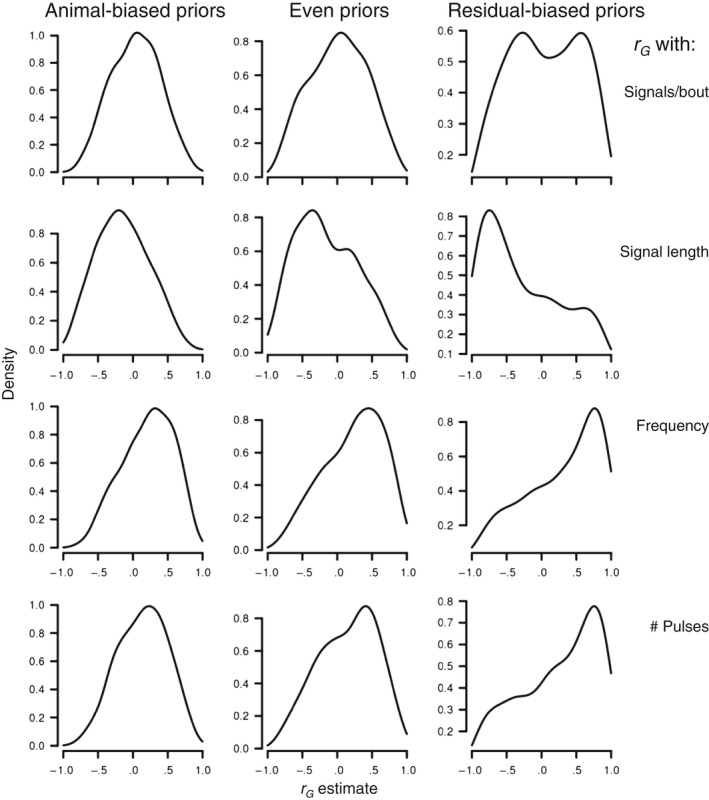
Posterior distributions of the estimates for the genetic correlation (*r*_G_) between female fecundity and male signal traits in *Enchenopa* in our rearing experiment, with the different priors used in the animal model

All *r*_G_ estimates by the animal model had 95% CIs that overlapped zero (Table 4). This result offers a rejection of the hypothesis that mate choice causes local adaptation (Table 1): Without support for prediction (ii), none of the other predictions can be supported. However, this could be due to the small sample of full‐sib families that our criteria for within‐family sample sizes allowed (*n *=* *16 families; see above) (cf. Sharma, Wilson, & Hosken, 2016). The mode of the posterior probability distributions of the animal model estimates was consistently at or near zero for only one signal trait (signals/bout) (Figure 4). For the other signal traits, the mode was consistently either positive (dominant frequency, number of pulses) or negative (signal length) across the different priors (Figure 4), suggesting that these correlations may be nonzero but that our analysis may be weakened by the small sample of families, which could represent support for prediction (ii). We therefore complemented our study with the below analyses, to seek a more robust test of the hypothesis.

**Table 4:** Animal model estimates of the genetic correlation (with 95% confidence interval) between *Enchenopa* female fecundity and male signal traits

	Animal‐based priors	Even priors	Residual‐based priors
Correlation with:			
Frequency	0.26 (−0.46 to 0.82)	0.30 (−0.56 to 0.92)	0.91 (−0.72 to 0.97)
Length	−0.25 (−0.79 to 0.56)	−0.25 (−0.89 to 0.60)	−0.90 (−0.97 to 0.79)
# pulses	0.15 (−0.54 to 0.77)	0.50 (−0.59 to 0.87)	0.87 (−0.82 to 0.97)
# signals/bout	−0.01 (−0.63 to 0.68)	0.12 (−0.76 to 0.73)	−0.19 (−0.82 to 0.94)

**Figure 5:**
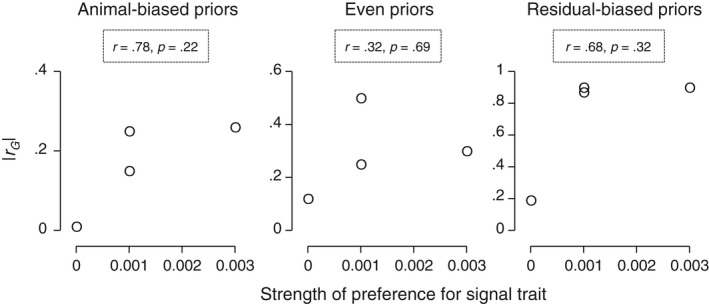
Relationship between the strength of mate preferences and the magnitude of the genetic correlation (*r*_G_) between female fecundity and the corresponding male signal trait in *Enchenopa* in our rearing experiment. We used the absolute value of *r*_G_ to focus on its magnitude. We show correlations for the *r*_G_ estimates obtained with the different priors used in the animal model

**Figure 6:**
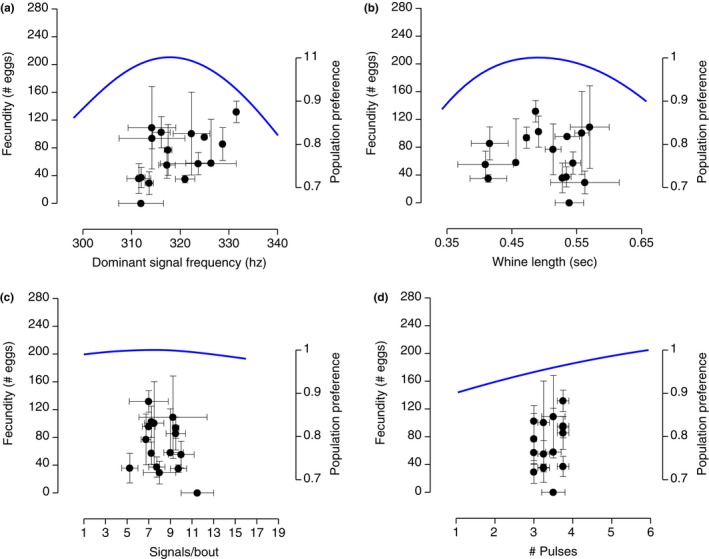
Comparison of the female fecundity~signal trait relationship (genotypic values obtained as family medians) and the population‐level female mate preference function for the signal trait. In each panel, the axes show a range corresponding to the mean ± 2 standard deviations. Symbols in black indicate family median values, and the error bars in black correspond to the 40th–60th percentiles. The curves in blue indicate the population‐level mate preference functions. (a) *r*_G_ between fecundity and signal frequency estimated with family median values was significant (*r *=* *.51, *p *=* *.042, *n *=* *16). There was no indication of curvilinearity (quadratic fit on signal frequency: *F*_2,13_ = 2.33, *p *=* *.14) and thus no match with the mate preference. (b) *r*_G_ between fecundity and signal length estimated with family median values was not significant (*r *= −.02, *p *=* *.95, *n *=* *16). There was also no indication of curvilinearity (quadratic fit: *F*_2,13_ = 0.28, *p *=* *.76), so that the relationship would not have matched the mate preference. (c) *r*_G_ between fecundity and the number of signals/bout estimated with family median values was not significant (*r *= −.30, *p *=* *.25, *n *=* *16). The test for curvilinearity was marginally significant (quadratic fit: *F*_2,13_ = 3.22, *p *=* *.07) but would not in any case result in genotypes associated with high fecundity being favored by the mate preference. (d) *r*_G_ between fecundity and then number of pulses was not significant (*r *=* *.26, *p *=* *.36, *n *=* *16), and there was no indication of curvilinearity (quadratic fit: *F*_2,13_ = 1.20, *p *=* *.33). Additionally, the range of genotypic values for pulse number was so narrow that it would not allow the mate preference to favor genotypes associated with high fecundity

We found correlations of mostly large effect size between the strength of mate preferences and the magnitude of |*r*_G_| between female fecundity and the signal traits (Figure 5). This lends support to prediction (iii) (Table 1). Additionally, we found a significant *r*_G_ estimate of large effect size between female fecundity and male signal frequency when we used family median values (Figure 6). This would support predictions (ii)–(iv) (Table 1), because signal frequency is the signal trait for which female mate preferences are strongest, and the signal trait that is most distinctive among the species in the *E. binotata* complex (Cocroft et al., 2010; Rodríguez et al., 2006).

Nevertheless, the shape of the fecundity~signal frequency relationship did not match the shape of the population mate preference, which is curvilinear and favors signal frequencies that are not associated with the highest fecundity values (Figure 6). This therefore fails to support predictions (v)–(vii) (Table 1). The estimates for *r*_G_ between family median values for female fecundity and the other signal traits were small to medium in effect size and nonsignificant, and would also not match the shape of the corresponding population mate preferences (Figure 6).

**Figure 7:**
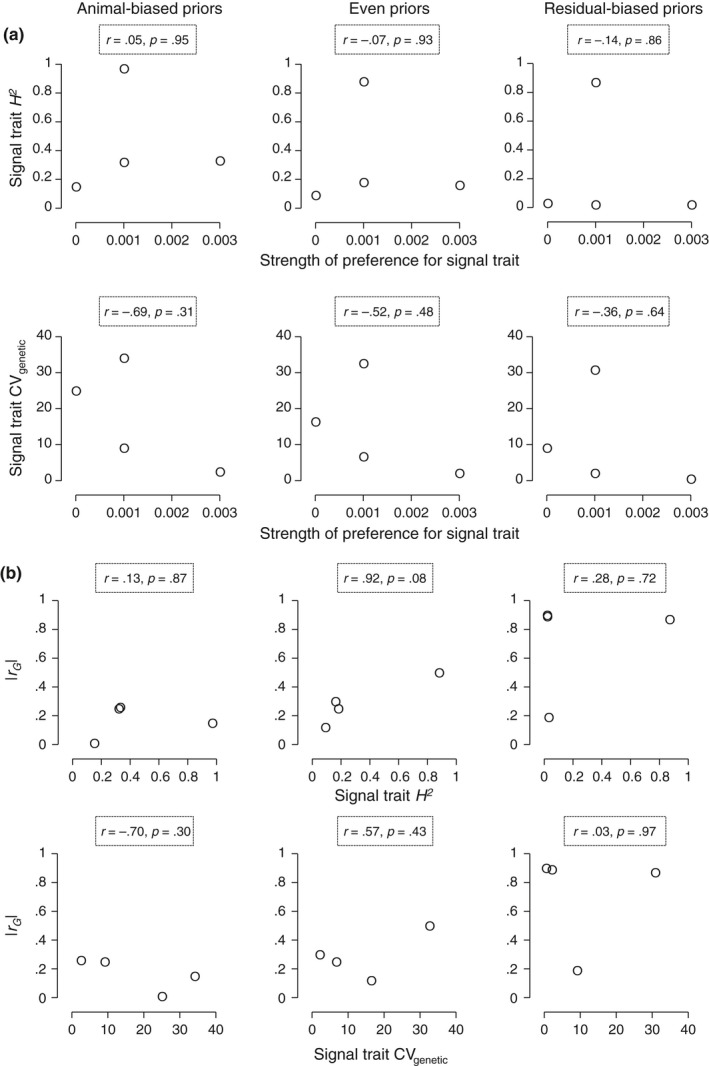
Analysis of potentially confounding factors in our test for genetic correlations between female fecundity and male signal traits. (a) Relationship between the strength of mate preferences and the amount of genetic variation in signal traits, measured as *H*_2_ or CV_genetic_ (see text). (b) Relationship between the amount of genetic variation in signal traits (measured as *H*_2_ or CV_genetic_) and the magnitude of *r*_G_ (absolute value) between female fecundity and the corresponding signal trait

In the corollary analyses, we found mixed results for the correlations between the strength of mate preferences and the amount of genetic variation in signal traits. Correlations with signal trait *H* ^2^ were very weak and of variable sign (Figure 7a). But, correlations with signal trait CV_genetic_ were of mostly large effect size and consistently negative (Figure 7a). These results thus leave some possibility that selection due to the mate preferences has eroded genetic variation in signal traits in such a way that it might limit the potential for the presence of fecundity~signal genetic correlations.

Results for the correlations between the amount of genetic variation in signal traits and the magnitude of *r*_G_ between female fecundity and the signal traits were also mixed. Correlations between signal trait *H* ^2^ and *r*_G_ were mostly weak, although consistently positive (Figure 7b). And, correlations between signal trait CV_genetic_ and *r*_G_ were mostly strong but of varying sign (Figure 7b). Thus, there is some suggestion that the amount of genetic variation in signal traits may limit the potential for fecundity~signal genetic correlations.

## 4 DISCUSSION

We tested the hypothesis that mate choice causes local adaptation such that, across environments, it can promote specialization and speciation (Cocroft et al., 2008; Lorch et al., 2003). We generated a suite of predictions reflecting the basic expectations that there should be a relationship between genetic variation in ecological performance and male advertisement signals, and that this relationship should be parallel to the mate preferences for those signal traits. A key feature of our test is the use of female fecundity as a measure of ecological performance, according to the rationale that for the hypothesis the most relevant component of performance relates to the expression in a female's daughters of genes borne by her male mating partner. We used a member of the *E. binotata* species complex of treehoppers, which allowed us to refine the predictions with background information about the signal traits that have the strongest mate preferences and that are the most divergent among closely related species (Cocroft et al., 2008, 2010; Rodríguez et al., 2006; current study).

Natural and sexual selection are involved in the process of speciation in the *E. binotata* complex, but divergent sexual selection due to mate choice is a main cause of signal evolution in the complex (Rodríguez et al., 2006; Sullivan‐Beckers & Cocroft, 2010). Consequently, if mate choice causes local adaptation and ecological divergence, genotypes with peak female performance should also have the male signal phenotypes favored by mate choice. Our results reject this hypothesis in two different ways. Animal model estimates for *r*_G_ between female fecundity and male signal traits all overlapped zero. However, this could reflect a sample size limitation in our rearing experiment, and thus not represent a robust rejection of the hypothesis. Further examination of our results did yield some apparent support for some of the predictions of the hypothesis: There were strong and positive correlations between the magnitude of *r*_G_ and the strength of the mate preferences for the corresponding signal traits. And, an alternative method using family median values did detect a strong value for *r*_G_ between female fecundity and a key signal trait—signal frequency, which has the strongest mate preferences and is the most divergent signal trait in the *E. binotata* complex; Rodríguez et al., 2006; Cocroft et al., 2010). These two results suggest that mate preferences could be selected to favor signal genotypes associated with high ecological performance. Nevertheless, this does not seem to have been the case: The signal frequencies that are favored by the population‐level mate preference are not associated with high fecundity.

Our rejection of the hypothesis that mate choice promotes local adaptation and ecological divergence is tentative because of the small sample size of full‐sib families that we were able to obtain, and we encourage further tests with other species. However, our findings suggest that signal‐preference divergence and ecological divergence may occur in parallel but separately during speciation. Even in such a case, there are various possible synergistic interactions between natural and sexual selection (Maan & Seehausen, 2011; Safran et al., 2013). For example, rapidly diverging signals and preferences may offer early prezygotic reproductive isolation, complemented eventually by postzygotic incompatibilities due to ecological specialization (Coyne & Orr, 2004). Nevertheless, our results suggest that the challenge for theory may lie in explaining how speciation can result from the joint but separate action of natural and sexual selection, rather than from a single process.

## CONFLICT OF INTEREST

None declared.

## AUTHOR CONTRIBUTIONS

KDFF: project conception and design, data acquisition, analysis and interpretation, writing; JTK: analysis and interpretation; DC: data acquisition; RLR: project conception and design, analysis and interpretation, writing.
